# Supplementary figures and images for: COVID-19 Recovery Time and Its Predictors among Hospitalized Patients in Designated Hospitals in the Madhesh Province of Nepal: A Multicentric Study
Source: Healthcare (Basel). 2024 Aug 24;12(17):1691. doi: 10.3390/healthcare12171691 (PMC11395077; doi:10.3390/healthcare12171691)

## Supplementary Materials:

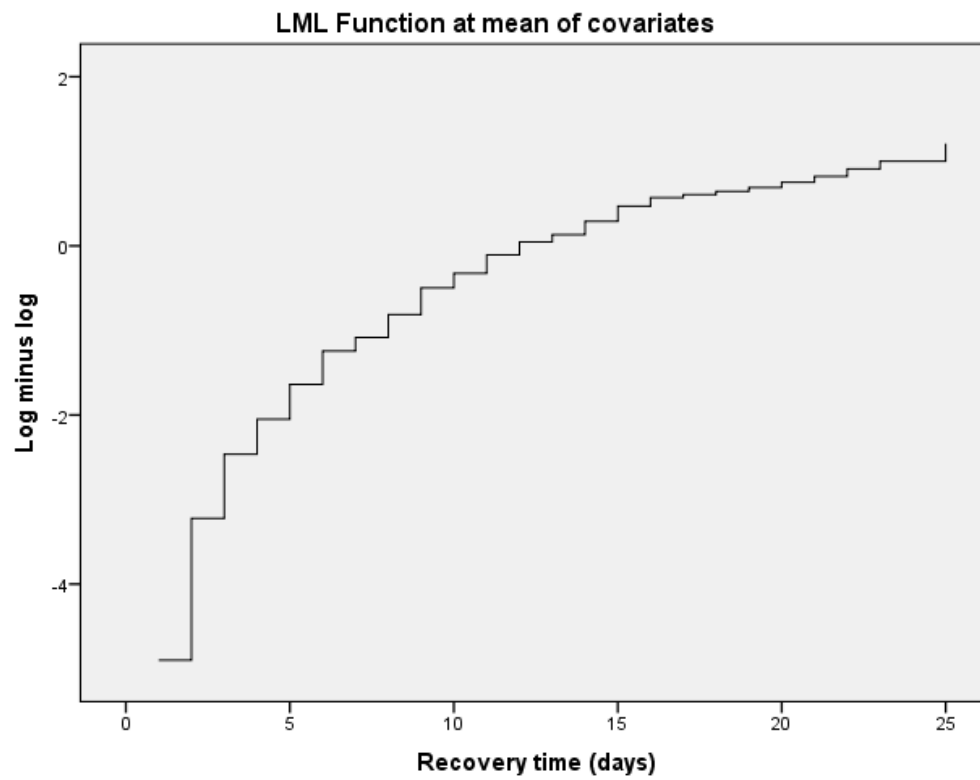

Figure S1. Log (-log) plot (Model-I)

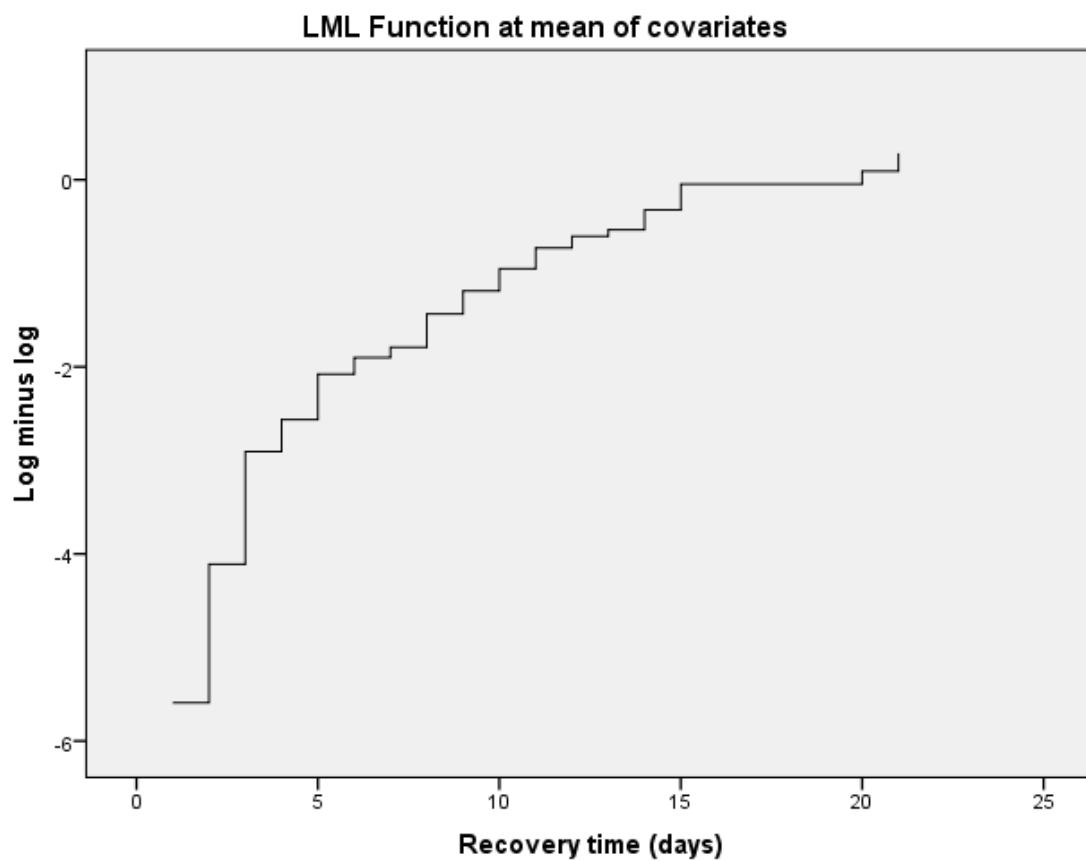

**Figure S2.** Log (-log) plot (Model-II)

Supplement: Supplementary file 1 [file healthcare-12-01691-s001.zip › healthcare-3166563-supplementary.pdf]
